# Supplementary material for: Predicting the replicability of social and behavioural science claims in COVID-19 preprints
Source: Nat Hum Behav. 2024 Dec 20;9(2):287–304. doi: 10.1038/s41562-024-01961-1 (PMC11860236; doi:10.1038/s41562-024-01961-1)
Supplement: Supplementary file 1 — Supplementary Materials 1–6. [file 41562_2024_1961_MOESM1_ESM.pdf]

nature human behaviour

Supplementary information

https://doi.org/10.1038/s41562-024-01961-1

Predicting the replicability of social and behavioural science claims in COVID-19 preprints

In the format provided by the authors and unedited

Supplementary Information
Predicting the replicability of social and behavioural science claims in COVID-19 preprints

November 15, 2024

Contents

1 Deviations from the preregistrations for the COVID-19 Preprint Replication Project 2

2 Replication outcomes and participants' predictions for the 35 replication studies 10

3 Replication outcomes and participants' predictions for research claims with at least 0.8 power with an alpha of 0.025 13

4 Figure SM5: Participants' best estimates for all 100 research claims 16

5 Bibliographic details of all 100 preprints 17

6 Questions used in the repliCATS process for eliciting judgements about replicability 25

Supplementary Materials 1

Deviations from the preregistrations for the COVID-19 Preprint Replication Project

Replicators produced a final report summarizing the methods and results of their study, a section of which documents the deviations between their preregistration and the study they conducted. Those sections are included below and organized by level of deviation. The text of each entry is drawn directly from the final reports, which are linked in Table SM1.

No deviations from the preregistration

Abouk & Heydari, 2020
Carrillo-Vega et al., 2020
de la Vega et al., 2020
Du et al., 2020
Erceg et al., 2020
Flesia et al., 2020
Gerhold, 2020 (analysis a)
Gerhold, 2020 (analysis b)
González-Marrón & Martínez-Sánchez, 2020
Hossain, 2020
Imhoff & Lamberty, 2020
Kuratani, 2020
Messner & Payson, 2020
Muto et al., 2020
Pennycook et al., 2020 (b)
Pfattheicher et al., 2020
Sala & Miyakawa, 2020
Seale et al., 2020
Šrol et al., 2020
Sternisko et al., 2020
Teovanović et al., 2020

Gerhold, 2020 (focal) [no preregistration]
Goh, 2020 (NA – deviations were not referenced in the final report)

Minor or superficial deviations from the preregistration

Bertin et al., 2020
None. Minor changes were made in relation to the pre-registered analysis script. This included correcting an error in the code for removing attention check fails and adding a section for generating a codebook.

Kachanoff et al., 2020
The preregistration code was updated with the following:

- Deidentification to ensure Prolific IDs were not shared.

- Code to help with the reproducibility (i.e., information on operating systems and versions).
- Excluded all test data because this is part of the raw data download.
- Excluded duplicates from participants taking the survey twice or opening and closing the survey several times within the same week.
- Matching participants when IDs were not recorded.
- Originally, we planned to hand score the attention check dog item. We did do that during the running of the study to know who to pay/keep track of participants. However, we updated the code in the analysis document to reflect our scoring using regular expressions to be clear and reproducible on what we thought reflected a correct answer.
- The section on allowable missing data was not included in the original code, as it was created with complete data. The section from our preregistration was pasted into the analysis section and code for those rules was added/updated to reflect our plan.

No deviations were made for the study delivery.

Kavanagh et al., 2020
There were no deviations from the preregistration plan, or changes to the preregistered script (except to remove the line limiting analysis to a random 5% subset of the data, and the aforementioned missing observation).

Malik et al., 2020
None. We commented out one line in the analysis script ("sample 5") which was responsible for randomly sampling 5% of the data for the preregistration, thus we performed the analysis on all of the data as planned.

Rothgerber et al., 2020
None. Minor changes were made to the pre-registered analysis script. This included correcting an error in the transformation of the social distancing variable (inverting scores prior to transformation, as reported by the authors of the original study) and adding code for creating a codebook for the final data.

Simione & Gnagnarella, 2020
The preregistration defined the demhealthcare as 1 for health care workers and 2 [not 0!] for non-health care workers. Hence, health care workers became the base category, which is the opposite of the original paper. Hence, this would change the 'expected' sign of the effect. In the script for this final report, I therefore recode demhealthcare to be consistent with the original paper and call it hc. The expected sign for hc is the same as for the health care worker dummy of the original paper. Note this changes the sign but not the significance and hence does not change that the overall outcome of the replication is unsuccessful.

Stanley et al., 2020
None. Minor changes were made to the pre-registered analysis script to update the coding of the free text entry item of the CRT measure. As noted in the original analysis script, lower case spelling and obvious typos of the 6th CRT item were counted as correct. As it states in the originally pre-data collection uploaded script, the entire code for correcting spelling errors was not shown in the script for the obvious reason that it depends on the exact types of spelling errors

in the final data. Therefore, the final analysis script was updated to include the final coding of the data for this question.

The updated final analysis script contains the analysis code for both stage 1 data collection, as well as stage 2 data (i.e. the combined data set from stage 1 and 2 collection).

Minor changes were also made to the sampling technique. In the pre-registration we had said that we would sample an additional 10% to account for attrition due to failing the attention check. When we ran data collection we implemented a 'real time sampling technique' which allowed us to automatically check whether a participant completed the attention check correctly.

Hence, the target analytic sample of 949 participants in stage 1 and the target analytic sample of 1191 in stage 2 data collection included only those participants who did not fail the attention check, as per the pre-registration. The only 'deviation' from the pre-registration therefore is that any excess sampling was not a mere estimation (the 10% indicated in the pre-registration) but an exact figure determined by the exact amount of participants who actually failed the attention check. As noted in the final analysis script, the target analytic sample data can be extracted from the data file subsetting to the 'end' variable value "complete". See further details in the analysis R script.

Wise et al., 2020
None. Minor changes were made to the pre-registered analysis script. This included adding code to enable labelling of the Stage 1 and Stage 2 dataset, and to allow appropriate filtering of over-quota participants as specified in Q15 of the preregistration (12 participants were eliminated from the Stage 1 dataset, six participants from Stage 2: in each case the participants were chosen on the basis of the quota they were in and the time they completed the survey as per the pre-registration).

Since Stage 1 failed to replicate and thus we had to proceed to Stage 2 data collection, code was also written to allow the combined Stage 1 and 2 dataset to be filtered to just Stage 1 such that Stage 1 analyses could be run from the combined dataset, but only on Stage 1 data.

We also added code to allow effect sizes (Cohen's f2 and standardised Beta) to be extracted, exact p-values (and other statistics e.g. t, SE, unstandardised beta) to be extracted and to allow the regression outputs to be tabulated and plotted.

Finally, we added code for creating a codebook for the final data and for outputting this final data to be uploaded publicly on OSF.

Wissmath et al., 2020
There was one deviation in the analysis script as the package to calculate the effect size associated with the ANOVA analysis (sjstats) was not installed.The new version of the script has been uploaded to the OSF project folder on 28 October 2020.

Substantive deviations from the preregistration

Al-Tammemi et al., 2020

The analyses were incorrectly coded in the preregistration, as it was unclear which variables were categorized from reviewing the paper. The motivation scale is presented as categorical, but was treated as continuous for purposes of analysis, which became clear after the analysis was examined. Additionally, the Kessler scale was made categorical. These were switched in the preregistered analysis, which was fixed in the updated analysis.

Other additions to the code include:

- Deidentification
- Addition of information provided by prolific participants
- Exclusion of test surveys that are included in the full data download
- Added a few clarifying lines to follow the code procedure (i.e., showed the number of values changing)
- Added a line determining final sample size

No deviations were made for the study delivery.

Bischetti et al., 2020

No deviations were made for the study delivery; however, we did make a few deviations for the payment during the study. As noted in our analysis script, the server we were running the study on crashed while many participants were taking the study. We paused the study immediately. We then went through the data to see what we had and notified participants they could finish the study if they desired (once the server was back up). We paid participants who completed at least half the study with half the incentive ($3.00) to account for the server crash. We then opened the rest of the slots back up to new participants on Prolific based on the money we had left to complete the study. Therefore, we recruited more than 1000 participants, but several only completed half the study. We used all data points in our analyses, as mixed models can account for this type of missing data.

Our analyses scripts were also updated:

- Reproducibility information about packages was added to the top of the script.
- We deidentified the data, as Prolific IDs may not be anonymous.
- We excluded data points from testers, as these rows are part of the raw data download from formr.
- We removed duplicates from the server crash.
- We added a state that a participant emailed us to add.
- We rescaled age from year of birth to age by subtracting 2021 – age.
- When writing the original code, we did not put the labels in the right order to get the COVID-verbal versus non-COVID-verbal. These levels were reordered.

A notable change from preregistration involves the model formula. We originally tried using the same model formula as the online provided script, albeit this model formula is not well described in the paper (i.e., it does not mention age). This model would not converge properly. We tried several different optimizers, which also would not converge. Therefore, we created a model with only one parameter and slowly added parameters until the model stopped working. It was

determined that the age random slopes were the problematic parameter, and it was removed. Otherwise, the random slopes and intercepts were included. Further, the paper reports what we think are marginal means from the model, but this code was not included in the original script (ours or theirs). We added emmeans to calculate these values.

Blagov, 2020
The additional collection was not pre-registered so this was a deviation from the pre-registration. However, this additional data collection was necessary in order to meet the target analytic sample size for Stage 1.

Columbus, 2020
One deviation in the analysis was that, due to confusion during the pre-registration process, the pre-registered analysis and dependent variable did not match the focal score claim. Therefore, prior to running the analysis, a decision was made to run the analysis instead using the Q9_1 dependent variable which actually measures future intentions to stockpile rather than past stockpiling behavior (which is what the pre-registered variable was measuring). As this variable was not binary, a binary logistic regression was not the correct form of analysis to use. Instead, a linear regression was used instead.

Pennycook et al., 2020 (a)
It turned out not to be possible to record the time elapsed between the start of the sharing task and the moment participants were shown a particular headline, as this function is not supported by Qualtrics. Instead, we therefore exported the viewing order of the randomised headlines, using the "Export viewing order data for randomized surveys" function. Third, we were unable to control for whether the treatment interacts with the nearest distance to the epicentre of a COVID-19 outbreak. Fourth, we conducted our analyses in STATA instead of R, following the original paper's analysis. Fifth, due to an error in the survey implementation, we were unable to include the pre-registered numeracy measures and rational versus intuitive style decision-making as correlates. Sixth, in the second stage of data collection, we recruited one more participant than necessary (1,583 versus the pre-registered 1,582).

Table SM1: Replication supplemental information

Paper	Project	Prereg	Report	Materials	Power	Data	Analysis
Abouk & Heydari, 2020	osf.io/nmzbh	osf.io/yma2c	osf.io/xk9rg	osf.io/vqa2y	osf.io/bq8g4	osf.io/tpd3j	osf.io/k3wbj
Al-Tammemi et al., 2020	osf.io/mqvyf	osf.io/mcrx3	osf.io/dmq92	osf.io/c3hzb	osf.io/w278s	osf.io/h7wd5	osf.io/38wku
Bertin et al., 2020	osf.io/y5kbe	osf.io/cuh8g	osf.io/w5edk	osf.io/92wku	osf.io/qejb3	osf.io/tgkwf	osf.io/up26w
Bischetti et al., 2020	osf.io/n2dhg	osf.io/xem4p	osf.io/aern6	osf.io/pcu9g	osf.io/qx59c	osf.io/shd6r	osf.io/u7evb
Blagov, 2020	osf.io/uzmtn	osf.io/w67pd	osf.io/92ujr	osf.io/5g27d	osf.io/9ev2t	osf.io/76ywq	osf.io/74eb8
Carrillo-Vega et al., 2020	osf.io/hpgvj	osf.io/k57gv	osf.io/8f2eg	osf.io/mj2qv	osf.io/qyr24	osf.io/jtqpn	osf.io/qrz6j
Columbus, 2020	osf.io/8f5by	osf.io/sk64a	osf.io/9ucsy	osf.io/8xe5r	osf.io/6h3zd	osf.io/7cz4j	osf.io/p67uy
de la Vega et al., 2020	osf.io/dy52c	osf.io/p354f	osf.io/8myjr	osf.io/29stx	osf.io/a5rhw	osf.io/btgcj	osf.io/2cs3a
Du et al., 2020	osf.io/da26y	osf.io/a6dev	osf.io/jz3ey	osf.io/m47tj	osf.io/vm6gs	osf.io/cfh7v	osf.io/6gjnz
Erceg et al., 2020	osf.io/z6mkt	osf.io/ydg9f	osf.io/9dr3g	osf.io/qjezn	osf.io/862an	osf.io/mn8rx	osf.io/r2qms
Flesia et al., 2020	osf.io/2px95	osf.io/6xk2q	osf.io/ux3sk	osf.io/8pqa6	osf.io/sf5t4	osf.io/vcwpq	osf.io/w48vf
Gerhold, 2020 (focal)**	osf.io/4rxgz		osf.io/qvrw3	osf.io/mxd5g		osf.io/rf65g	osf.io/dxey9
Gerhold, 2020 (analysis a)**	osf.io/a7h9n	osf.io/fbn9y	osf.io/7hn85	osf.io/x4sbm	osf.io/7ur2v	osf.io/6tckw	osf.io/fam8x
Gerhold, 2020 (analysis b)**	osf.io/2zre3	osf.io/c847x	osf.io/xkn7r	osf.io/42wmg	osf.io/mfe6t	osf.io/qch6a	osf.io/k8u75/
Goh, 2020	osf.io/3czus	osf.io/7qnw8	osf.io/gwz6s	osf.io/xdb72	osf.io/jmy7f	osf.io/vm7kw	osf.io/edqz9

González-Marrón & Martínez-Sánchez, 2020	osf.io/pr5jm	osf.io/wa8ux	osf.io/m7u5j	osf.io/29bep	osf.io/ju78w	osf.io/pdfbv	osf.io/twn7d
Hossain, 2020	osf.io/h6wg9	osf.io/jnhbz	osf.io/gt73n	osf.io/5tnqy	osf.io/anesy	osf.io/8gh6c	osf.io/emk2t
Imhoff & Lamberty, 2020	osf.io/9beuv	osf.io/ypfn3	osf.io/fmce6	osf.io/4cgh6	osf.io/q6k5r	osf.io/bkpcj	osf.io/vhd7k
Kachanoff et al., 2020	osf.io/ytuk9	osf.io/smcxg	osf.io/xpz8v	osf.io/y2qv5	osf.io/y6c57	osf.io/3cavs	osf.io/xshv6
Kavanagh et al., 2020	osf.io/gdv4x	osf.io/3uvyk	osf.io/4kmc6	osf.io/vhgc2	osf.io/zvdyr	osf.io/u94x3	osf.io/zx7dn
Kuratani, 2020	osf.io/kbm46	osf.io/5zk9v	osf.io/9v4ka	osf.io/bzkes	osf.io/wjuh5	osf.io/xf2b9	osf.io/3qg8a
Malik et al., 2020	osf.io/zd2uq	osf.io/u5z7m	osf.io/xr9mn	osf.io/wbxka	osf.io/asz97	osf.io/ebgqz	osf.io/t4qh5
Messner & Payson, 2020	osf.io/4589g	osf.io/qmxwh	osf.io/rc5ez	osf.io/5cg96	osf.io/jrfz2	osf.io/dnbzr	osf.io/dch27
Muto et al., 2020	osf.io/8uex5	osf.io/ywphm	osf.io/ub42t	osf.io/ew3f7	osf.io/ceag3	osf.io/j36ch	osf.io/7utmx
Pennycook et al., 2020 (a)	osf.io/rkfq5	osf.io/yp6dc, osf.io/btkwp	osf.io/upydg	osf.io/akv68	osf.io/rhvb9	osf.io/9jxnc	osf.io/abr62
Pennycook et al., 2020 (b)	osf.io/6ewha	osf.io/vra45	osf.io/37hmp	osf.io/u5qv9	osf.io/nydmu	osf.io/whjg5	osf.io/wsy63
Pfattheicher et al., 2020	osf.io/nv6a3	osf.io/6zxh8	osf.io/vk4br	osf.io/uhm5d	osf.io/sfyzd	osf.io/ef58n	osf.io/vn6wp
Rothgerber et al., 2020	osf.io/ky2wm	osf.io/kehx7	osf.io/xrqc2	osf.io/9m26p	osf.io/k8rqn	osf.io/qrasz	osf.io/sdjrz
Sala & Miyakawa, 2020	osf.io/6zjdh	osf.io/rwxs9	osf.io/rkd75	osf.io/5qj4d	osf.io/b2ndp	osf.io/qtwvd	osf.io/xsyu2

Seale et al., 2020	osf.io/xr8gz	osf.io/3gdhp	osf.io/mxvc3	osf.io/3kx8r	osf.io/me65h	osf.io/p5dqt	osf.io/58rzn
Simione & Gnagnarella, 2020	osf.io/md5pu	osf.io/jn67f	osf.io/muezk	osf.io/tkz38	osf.io/7ejn8	osf.io/ty5b6	osf.io/cp3fn
Šrol et al., 2020	osf.io/cqxyh	osf.io/8xn5b	osf.io/pyb8z	osf.io/3nr7x	osf.io/guekr	osf.io/8zb9d	osf.io/7z8pk
Stanley et al., 2020	osf.io/cbknq	osf.io/6c5g9	osf.io/m7x3r	osf.io/4c9ua	osf.io/4eh7d	osf.io/tfvqs	osf.io/nvpeh
Sternisko et al., 2020	osf.io/uscaj	osf.io/cr5xv	osf.io/abu46	osf.io/ex2d7	osf.io/gua49	osf.io/r8kgt	osf.io/cgeju
Teovanović et al., 2020	osf.io/5vgjw	osf.io/dnqwy	osf.io/8ghzj	osf.io/zyc8e	osf.io/dmuy7	osf.io/h7kge	osf.io/dp8xw
Wise et al., 2020	osf.io/fypm4	osf.io/2qmh5	osf.io/j4s6p	osf.io/b2x4v	osf.io/z6nmg	osf.io/q5g3r	osf.io/kq4vj
Wissmath et al., 2020	osf.io/fjbvp	osf.io/d9ekr	osf.io/g8jnk	osf.io/yxfdh	osf.io/zksb9	osf.io/25m8q	osf.io/prn9e

The OSF columns are the main study page; preregistration; final report; study materials; power analysis; study data; and code.
**Gerhold, 2020 was replicated in two independent studies relying on different secondary data sources, represented by ‘analysis a’ and ‘analysis b’. A combined analysis drawing on both studies’ data represents the single replication outcome for this paper (‘Gerhhold focal’) that was used for determining prediction accuracy with results reported in Table 1.

Supplementary Materials 2

Replication outcomes and participants' predictions for the 35 replication studies

These analyses include 6 additional secondary data replications which were deemed underpowered and excluded from the main text of the paper. Among the 19 (total number of secondary data replication studies), 9 were replicated (47%), which produced an overall replication rate of 57% when including the 11 out of 16 new data replications (69%). Taken together, experienced participants predicted a replication rate of 62.6% in Round 1 and 62.4% in Round 2. Beginners predicted a replication rate of 66.5% in Round 1 and a slightly higher rate of 68.5% in Round 2 (Figure SM1).

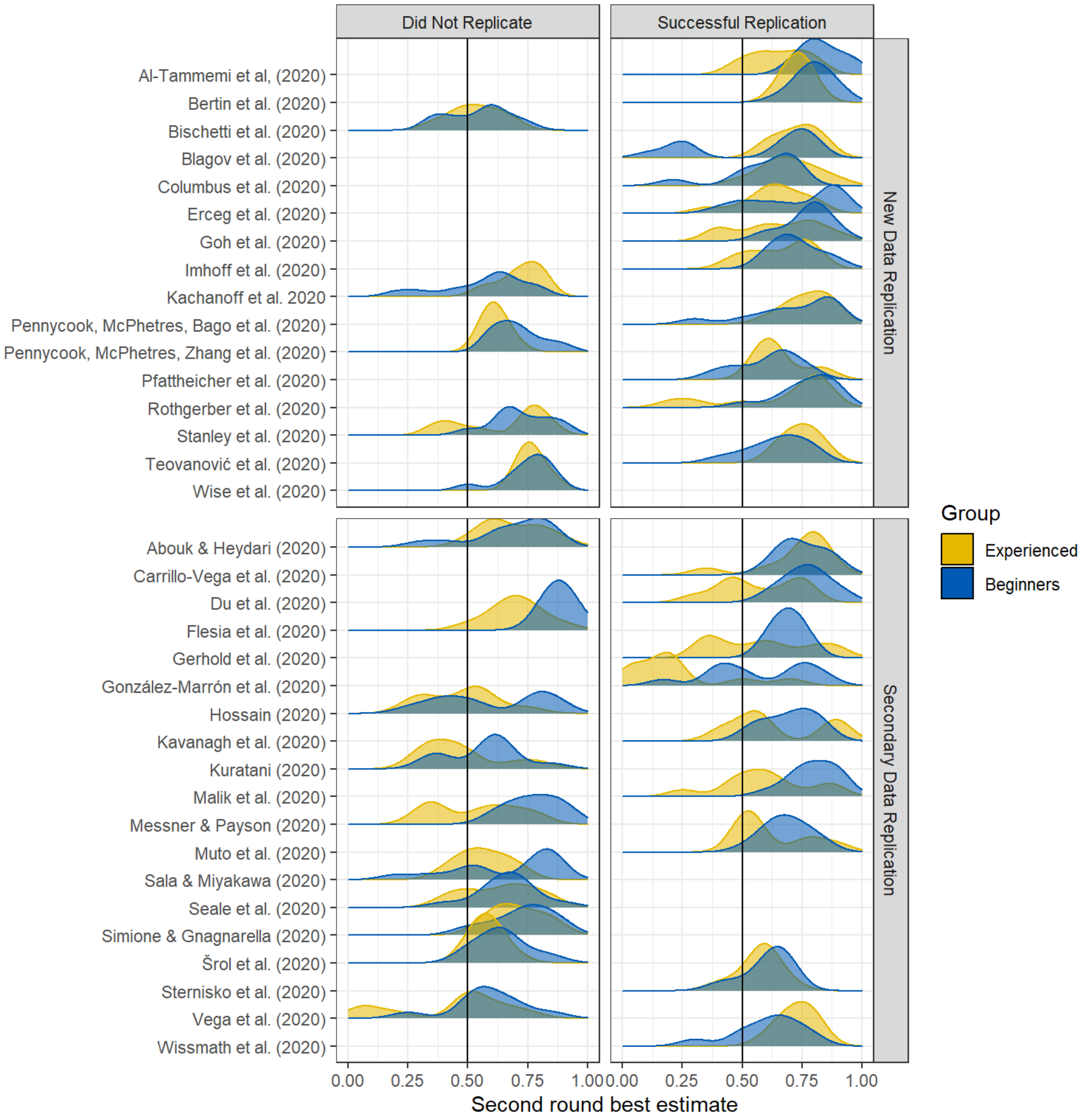

Figure SM1: Smoothed distribution of participants' best estimates for each of the 35 known-outcome research claims, organised by type of replication (new or secondary data) and success (did or did not replicate). Experienced participants are shown in yellow and beginners in blue.

Accuracy on claims with known-replication outcomes

Both beginners and experienced participants' judgments are consistent with some predictive ability, but prediction accuracy is not significantly better than chance for either group. The 95% confidence intervals of both beginners and experienced participants overlap .5 (Figure SM2) and participants' predictions are only weakly correlated to the outcome of the replication (r=.14 for beginners and r=.17 for experienced participants).

Differences between beginners and experienced participants

Against our pre-registered hypothesis, we observed no significant difference between the error-based accuracy of beginner and experienced participants' initial judgements (Round 1), at either the individual ($\hat{\beta}$=-0.011, SE=0.0174, t(731.000)=-0.63, p=.529, Figure SM2.a), or group level ($\hat{\beta}$=-0.025, SE=0.0265, t(137.000)=-0.935, p=.351, Figure SM2.c). Likewise, error-based accuracy after discussion (Round 2) did not differ between them, at either the individual ($\hat{\beta}$=0.0002, SE=0.0167, t(717.000)=0.017, p=.986), or group level ($\hat{\beta}$=-0.011, SE=0.0307, t(137.000)=-0.347, p=.729). There were also no differences between beginners and experienced participants in terms of classification accuracy in Round 1 ($\hat{\beta}$=0.016, SE=0.0358, t(351.171)=0.446, p=.656) or in Round 2 ($\hat{\beta}$=0.060, SE=0.0352, t(341.108)=1.719, p=.087, Figure SM2.b). At the group level, experienced participants correctly classified 61% and beginners correctly classified 57% of the claims they assessed in Round 1. In Round 2, beginners' classification accuracy increased to 63%, and experienced participants' decreased to 57% of claims.

Despite the similarities between the two groups in overall accuracy, beginners were more likely to shift their best estimates in the right direction after discussion, thus improving their accuracy more than experienced participants. On error-based accuracy, there was a notable but non-significant difference between them in the magnitude of the shift at both the individual and group level ($\hat{\beta}$=0.015, SE=0.010, t(718)=1.592, p=.112, and $\hat{\beta}$=0.014, SE=0.008, t(138)=1.666, p=.098, respectively). On classification accuracy, we observed a very small difference between beginner and experienced participants' shifts at the individual level ($\hat{\beta}$=0.051, SE=0.022, t(341.994)=2.344, p=.020).

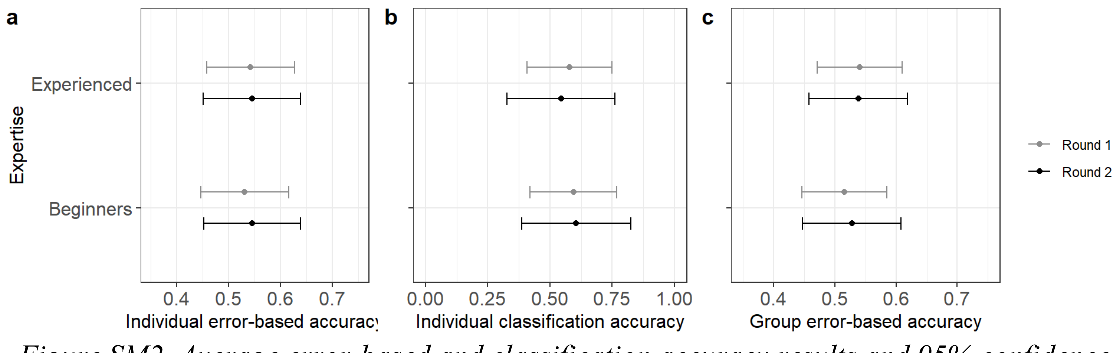

Figure SM2. Average error-based and classification accuracy results and 95% confidence intervals for both individuals and groups of beginners and experienced participants

Predictors of accuracy

None of the participants' characteristics we tested were reliably correlated with judgement accuracy. Correlations were weak between classification accuracy and metaresearch experience (r=-0.0553 and r=-0.059 for Round 1 and 2, respectively), experience with preregistration (r=-0.021 and r=-0.022), quiz total scores (r=0.078 and r=-0.013), quiz statistic

questions (r =0.111 and r=0.004), quiz replication questions (r=-0.007 and r=0.004), quiz questions on questionable research practices (r=-0.101 and r=-0.155).

Accuracy of market prices and comparison between elicitation methods

As we did for the results of the N=29 replications in the main manuscript, we report on four methods for predicting replicability in the prediction markets: the p-value based initial prediction market price, the final prediction market price, the survey means, and surrogate-scoring based aggregated means that only included forecasts of the 5 top-ranked forecasters. Average forecast, average error-based accuracy (including 95% CI), classification accuracy and the Spearman correlation between forecast and outcome (including p-value) are shown in Table SM2.

Table SM2. Results from the replication markets project

**Assessment Method**	**Average Forecast**	**Average error-based accuracy [95% CI]**	**Average classification accuracy**	**Correlation between forecast and outcome**	**p-value**
P-value based	.60	.61 [.53-.69]	.71	0.42	0.013
Final market price	.62	.57 [.5-.64]	.60	0.34	0.047
Survey means	.65	.58 [.51-.65]	.63	0.51	0.002
Surveys, SSR aggregated	.66	.60 [.51-.69]	.66	0.44	0.008

All other results reported in the main text of the paper remain the same.

Supplementary Materials 3

Replication outcomes and participants' predictions for research claims with ≥0.8 power with an alpha of 0.025

These analyses exclude 5 replications which were deemed to be underpowered at an alpha level of 0.025. Of the 24 included replications, 14 were based on new data collection by the replication team ('new data replications') and 10 were replications based on a secondary data source different from the original data ('secondary data replications'; see Methods). Among the former, 10 were successfully replicated (71%), while among the latter, 6 replicated (60%), for an overall replication rate of 67%. Experienced participants predicted a replication rate of 64.3% in Round 1 and 64.2% in Round 2. Beginners predicted a replication rate of 66.4% in Round 1 and a slightly higher rate of 69.1% in Round 2 (Figure SM3).

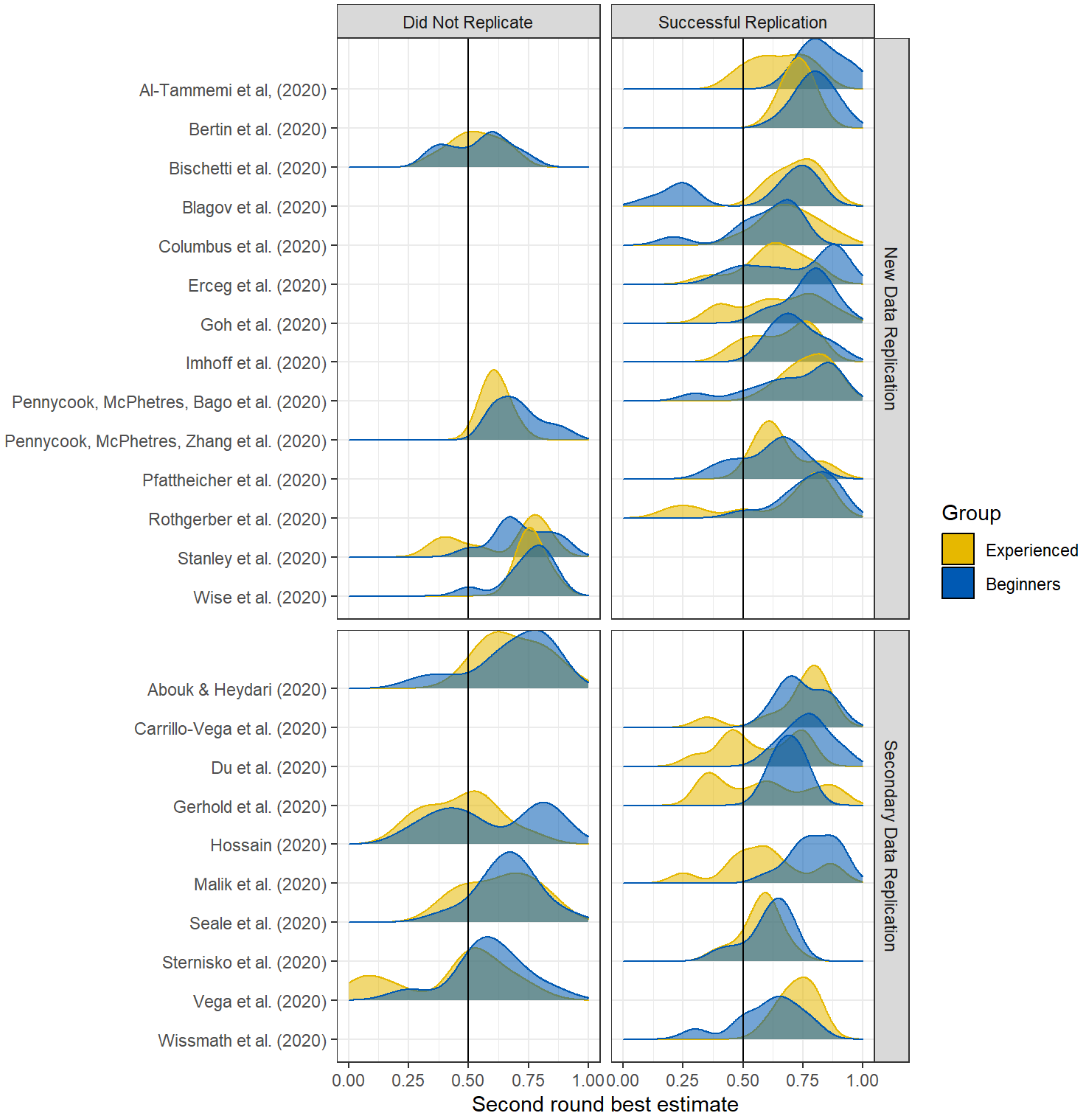

Figure SM3: Smoothed distribution of participants' best estimates for each of the 24 known-outcome research claims with ≥0.8 power with an alpha of 0.025, organised by type of replication (new or secondary data) and success (did or did not replicate). Experienced participants are shown in yellow and beginners in blue.

4. Accuracy on claims with known-replication outcomes

We used two measures of accuracy in this study: error-based accuracy and classification accuracy. We calculated error-based accuracy as 1 minus the absolute difference between an estimate (0-1) and the replication outcome (0 for failed replications, 1 for successful replications). Classification accuracy was calculated as the proportion of estimates on the correct side of .5 (estimate $\geqslant$ .5 for successful replications, estimate < .5 for failed replications) for a paper's replication outcome. Error-based accuracy was calculated at the individual and group level (i.e. based on the pooled estimates within a given group).

The judgments of both beginners and more experienced participants are consistent with some predictive ability, with both groups performing better than chance (i.e., their 95% confidence intervals do not overlap .5) on most accuracy measures (Figure SM4). However, participants' predictions are only weakly correlated to the outcome of the replication (r=.29 for beginners and r=.38 for experienced participants). All models used are described in the Methods section (Table M4).

Differences between beginners and experienced participants

Against our pre-registered hypothesis, we observed no significant difference between the error-based accuracy of beginners' and more experienced participants' initial judgements (Round 1), at either the individual ($\hat{\beta}$=-0.004, SE=0.021, t(496.00)=-0.202, p=0.840, Figure SM4.a), or group level ($\hat{\beta}$=-0.038, SE=0.030, t(94)=-1.235, p=0.22, Figure SM4.c). Likewise, error-based accuracy after discussion (Round 2) did not differ between the two samples, at either the individual ($\hat{\beta}$=0.011, SE=0.020, t(486)=0.556, p=0.579), or group level ($\hat{\beta}$=-0.026, SE=0.036, t(94)=-0.736, p=0.463). There were also no differences between beginners and more experienced participants in terms of classification accuracy in Round 1 ($\hat{\beta}$=0.050, SE=0.044, t(305.91)=1.135, p=0.257) or Round 2 ($\hat{\beta}$=0.082, SE=0.044, t(298.37)=1.862, p=0.064, Figure SM4.b).

At the group level, experienced participants correctly classified 71% and beginners correctly classified 58% of the claims they assessed in Round 1. Both performed better on the new-data replications (75% for experienced participants, 64% for beginners), compared to the secondary-data replications (65% for experienced participants, 50% for beginners). In Round 2, classification accuracy was near-identical among experienced (69%) and beginner participants (67%), which represented a decrease for the former, but an increase for the latter. The experienced participants' classification accuracy was unchanged in Round 2 for new-data replications (75%), but lower for secondary-data replications (60%), whereas the beginners' classification accuracy improved for secondary-data replications (70%) in Round 2 and remained the same for new-data replications (64%).

On classification accuracy, beginners did not shift their best estimates in the right direction after discussion at a higher rate than experienced participants ($\hat{\beta}$=0.040, SE=0.028, t(298.94)=1.436, p=0.152). On error-based accuracy, the difference between beginners and more experienced participants in the magnitude of the shift at both the individual and group level was also not significant ($\hat{\beta}$=0.018, SE=0.012, t(487)=1.497, p=0.135, and $\hat{\beta}$=0.011, SE=0.011, t(94)=1.008, p=0.316, respectively).

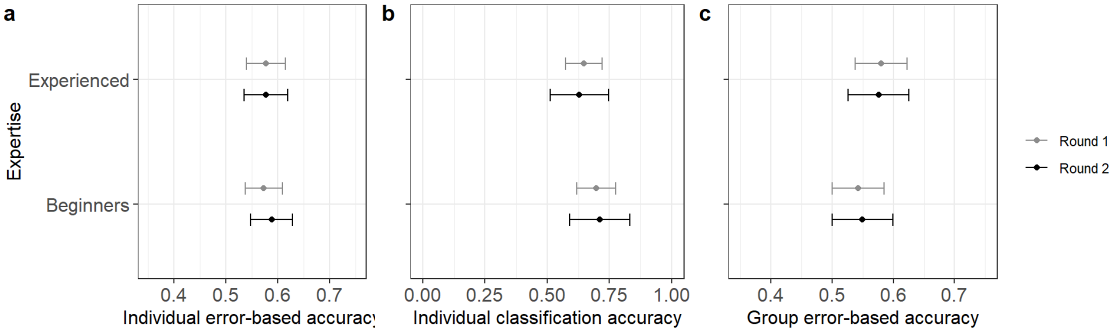

Figure SM4. Average error-based and classification accuracy results and 95% confidence intervals for both individuals and groups of beginners and experienced participants

Predictors of accuracy

None of the participants' characteristics we tested were reliably correlated with judgement accuracy in this study. We did not detect any notable correlations between classification accuracy and metaresearch experience (r=-0.037 and r=-0.066 for Round 1 and 2, respectively), experience with preregistration (r=-0.041 and r=-0.068), quiz total scores (r=0.063 and r=-0.047), quiz statistic questions (r =0.093 and r=-0.032), quiz replication questions (r=0.003 and r=-0.003), quiz questions on questionable research practices (r=-0.082 and r= -0.143).

Accuracy of market prices and comparison between elicitation methods

As we did for the results of the N=29 replications in the main manuscript, we report on four methods for predicting replicability in the prediction markets: the p-value based initial prediction market price, the final prediction market price, the survey means, and surrogate-scoring based aggregated means that only included forecasts of the 5 top-ranked forecasters. Average forecast, average error-based accuracy (including 95% CI), classification accuracy and the Spearman correlation between forecast and outcome (including p-value) are shown in Table SM3.

Table SM3. Results from the replication markets project.

**Assessment Method**	**Average Forecast**	**Average error-based accuracy [95% CI]**	**Average classification accuracy**	**Correlation between forecast and outcome**	**p-value**
P-value based	.60	.68 [.59-.77]	.83	0.63	0.001
Final market price	.62	.63 [.55-.71]	.71	0.56	0.004
Survey means	.65	.63 [.55-.71]	.75	0.63	0.001
Surveys, SSR aggregated	.66	.67 [.57-.77]	.79	0.56	0.005

All other results reported in the main text of the paper remain the same.

Supplementary Materials 4

Figure SM5: Participants' best estimates. Smoothed distribution of participants' best estimates for all 100 research claims. Experienced participants are shown in yellow, and beginners in blue.

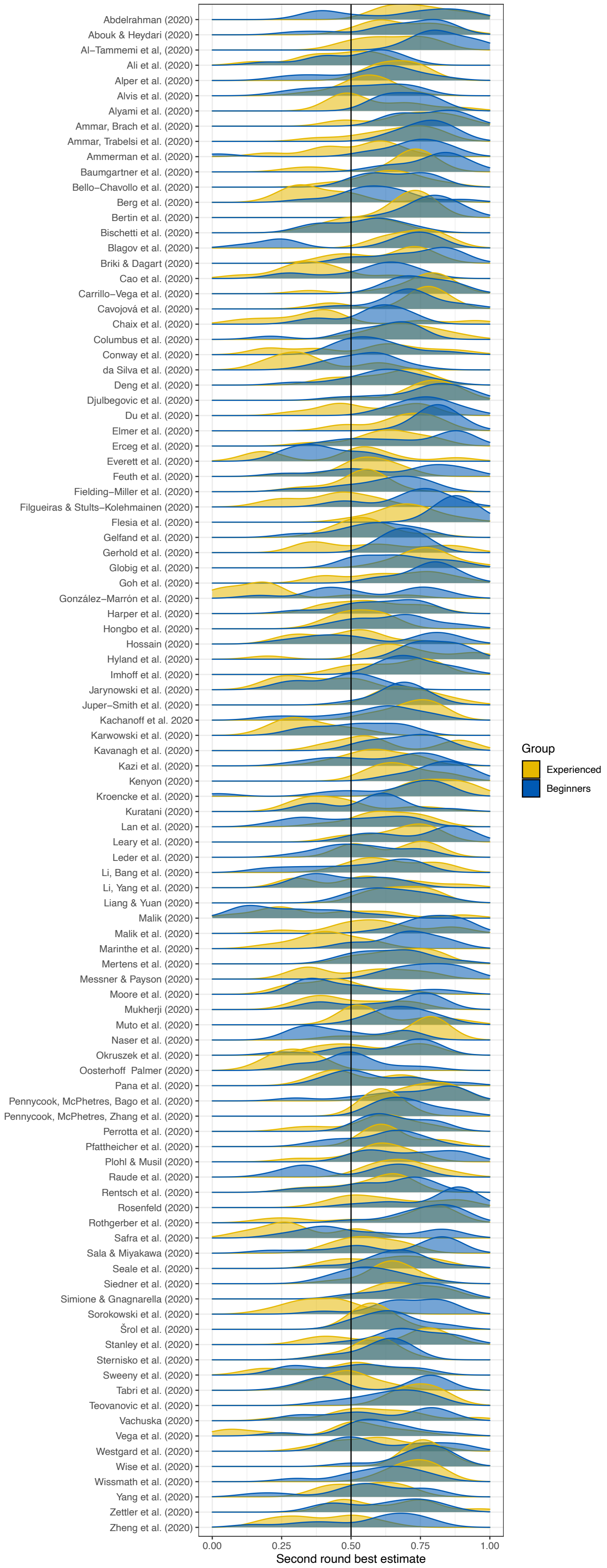

Supplementary Materials 5

Bibliographic details of all 100 preprints

Abdelrahman, M. K. (2020). *Personality Traits, Risk Perception, and Protective Behaviors of Arab residents of Qatar during the COVID-19 pandemic*. doi:10.31234/osf.io/6g7kh

Abouk, R., & Heydari, B. (2020). *The Immediate Effect of COVID-19 Policies on Social Distancing Behavior in the United States*. doi:10.1101/2020.04.07.20057356

Al-Tammemi, A. B., Akour, A., & Alfalah, L. (2020). *Is it Just About Physical Health? An Online Cross-Sectional Study Exploring the Psychological Distress Among University Students in Jordan in the midst of COVID-19 Pandemic*. doi:10.1101/2020.05.14.20102343

Alper, S., Bayrak, F., & Yilmaz, O. (2020). *Psychological Correlates of COVID-19 Conspiracy Beliefs and Preventive Measures: Evidence from Turkey*. doi:10.31234/osf.io/mt3p4

Alvis, L., Douglas, R., Shook, N., & Oosterhoff, B. (2020). *Associations between Adolescents' Prosocial Experiences and Mental Health During the COVID-19 Pandemic*. doi:10.31234/osf.io/2s73n

Alyami, H. S., Naser, A. Y., Dahmash, E. Z., Alyami, M. H., & Alyami, M. S. (2020). *Depression and anxiety during the COVID-19 pandemic in Saudi Arabia: a cross-sectional study*. doi:10.1101/2020.05.09.20096677

Ammar, A., Brach, M., Trabelsi, K., Chtourou, H., Boukhris, O., Masmoudi, L., … Hoekelmann, A. (2020). *Effects of COVID-19 home confinement on physical activity and eating behaviour Preliminary results of the ECLB-COVID19 international online-survey*. doi:10.1101/2020.05.04.20072447

Ammar, A., Trabelsi, K., Brach, M., Chtourou, H., Boukhris, O., Masmoudi, L., … Hoekelmann, A. (2020). *Effects of home confinement on mental health and lifestyle behaviours during the COVID-19 outbreak: Insight from the \textquotedblleftECLB-COVID19\textquotedblright multi countries survey*. doi:10.1101/2020.05.04.20091017

Ammerman, B. A., Burke, T. A., Jacobucci, R., & McClure, K. (2020). *Preliminary Investigation of the Association Between COVID-19 and Suicidal Thoughts and Behaviors in the U.S.* doi:10.31234/osf.io/68djp

Baumgartner, M. T., Lansac-Tôha, F. M., Coelho, M. T. P., Dobrovolski, R., & Diniz-Filho, J. A. F. (2020). *Social distancing and movement constraint as the most likely factors for COVID-19 outbreak control in Brazil*. doi:10.1101/2020.05.02.20088013

Bello-Chavolla, O. Y., González-Díaz, A., Antonio-Villa, N. E., Fermín-Martínez, C. A., Márquez-Salinas, A., Vargas-Vázquez, A., … Gutiérrez-Robledo, L. M. (2020). *Unequal impact of structural health determinants and comorbidity on COVID-19 severity and*

lethality in older Mexican adults: Looking beyond chronological aging. doi:10.1101/2020.05.12.20098699

Berg, M. K., Yu, Q., Salvador, C. E., Melani, I., & Kitayama, S. (2020). *Mandated Bacillus Calmette-Guérin (BCG) vaccination predicts flattened curves for the spread of COVID-19*. doi:10.1101/2020.04.05.20054163

Bertin, P., Nera, K., & Delouvée, S. (2020). *Conspiracy Beliefs, Rejection of Vaccination, and Support for hydroxychloroquine: A Conceptual Replication-Extension in the COVID-19 Pandemic Context*. doi:10.31234/osf.io/rz78k

Bischetti, L., Canal, P., & Bambini, V. (2020). *Funny but aversive: A large-scale survey of the emotional response to Covid-19 humor in the Italian population during the lockdown*. doi:10.31234/osf.io/efk93

Blagov, P. S. (2020). *Adaptive and Dark Personality Traits in the Covid-19 Pandemic: Predicting Health-behavior Endorsement and the Appeal of Public-health Messages*. doi:10.31234/osf.io/chgkn

Briki, W., & Dagot, L. (2020). *\textquotedblleftLiberate!\textquotedblright \textendash Republicans Are More Willing to Get Back to Social Life Because They Are Less Scared of COVID-19*. doi:10.31234/osf.io/thuwd

Cao, S., Qi, Y., Huang, Q., Wang, Y., Han, X., Liu, X., & Wu, H. (2020). *Emerging infectious outbreak inhibits pain empathy mediated prosocial behaviour*. doi:10.31234/osf.io/5p8kd

Carrillo-Vega, M. F., Salinas-Escudero, G., Garcia-Peña, C., Gutierrez-Robledo, L. M., & Parra-Rodriguez, L. (2020). *Early estimation of the risk factors for hospitalisation and mortality by COVID-19 in Mexico*. doi:10.1101/2020.05.11.20098145

Cavojova, V., Šrol, J., & Mikušková, E. B. (2020). *How scientific reasoning correlates with health-related beliefs and behaviors during the COVID-19 pandemic?* doi:10.31234/osf.io/tfy5q

Chaix, B., Delamon, G., Guillemasse, A., Brouard, B., & Bibault, J.-E. (2020). *Psychological Distress during the COVID-19 pandemic in France: a national assessment of at-risk populations*. doi:10.1101/2020.05.10.20093161

Chevallier, C., Sijilmassi, A., & Safra, L. (2020). *Disease, perceived infectibility and threat reactivity: a COVID-19 study*. doi:10.31234/osf.io/jy97m

Columbus, S. (2020). *Honesty-Humility, beliefs, and prosocial behaviour: A test on stockpiling during the COVID-19 pandemic*. doi:10.31234/osf.io/8e62v

Conway, L. G., Woodard, S. R., Zubrod, A., & Chan, L. (2020). *Why are Conservatives Less Concerned about the Coronavirus (COVID-19) than Liberals? Comparing Political, Experiential, and Partisan Messaging Explanations*. doi:10.31234/osf.io/fgb84

da Silva, J. G., Silva, C. S., Alexandre, B., & Morgado, P. (2020). *Health literacy of inland population in the mitigation phase 3.2. of COVID-19\textquotesingles pandemic in Portugal - a descriptive cohort study*. doi:10.1101/2020.05.11.20098061

Deng, S., Wang, S., Xie, P., Chao, Y., & Zhu, J. (2020). *Perceived Severity of COVID-19 and Post-pandemic Consumption Willingness: The Roles of Boredom and Sensation-Seeking*. doi:10.31234/osf.io/ch72a

Djulbegovic, B., Weiss, D. J., & Hozo, I. (2020). *Evaluation of the US governors decision when to issue stay-at-home orders [Running title: When to shut down: the Weber-Fechner law describes the timing of U. S. governors decisions to issue stay-at-home orders]*. doi:10.1101/2020.05.14.20093633

Du, H., Yang, J., King, R. B., Yang, L., & Chi, P. (2020). *Preprint of 'COVID-19 Increases Online Emotional and Health-Related Searches'*. doi:10.31234/osf.io/5gskw

Elmer, T., Mepham, K., & Stadtfeld, C. (2020). *Students under lockdown: Comparisons of students' social networks and mental health before and during the COVID-19 crisis in Switzerland*. doi:10.31234/osf.io/ua6tq

Erceg, N., Ružojčić, M., & Galic, Z. (2020). *Misbehaving in the Corona Crisis: The Role of Anxiety and Unfounded Beliefs*. doi:10.31234/osf.io/cgjw8

Everett, J. A. C., Colombatto, C., Chituc, V., Brady, W. J., & Crockett, M. (2020). *The effectiveness of moral messages on public health behavioral intentions during the COVID-19 pandemic*. doi:10.31234/osf.io/9yqs8

Feuth, T., Saaresranta, T., Karlsson, A., Valtonen, M., Peltola, V., Rintala, E., & Oksi, J. (2020). *Is sleep apnoea a risk factor for Covid-19? Findings from a retrospective cohort study*. doi:10.1101/2020.05.14.20098319

Fielding-Miller, R. K., Sundaram, M. E., & Brouwer, K. (2020). *Social determinants of COVID-19 mortality at the county level*. doi:10.1101/2020.05.03.20089698

Flesia, L., Monaro, M., Mazza, C., Fietta, V., Colicino, E., Segatto, B., & Roma, P. (2020). *Predicting Perceived Stress Related to the Covid-19 Outbreak through Stable Psychological Traits and Machine Learning Models*. doi:10.31234/osf.io/yb2h8

Gelfand, M., Jackson, J. C., Pan, X., Nau, D. S., Dagher, M. M., van Lange, P., & Chiu, C.-Y. (2020). *The Importance of Cultural Tightness and Government Efficiency For Understanding COVID-19 Growth and Death Rates*. doi:10.31234/osf.io/m7f8a

Gerhold, L. (2020). *COVID-19: Risk perception and Coping strategies*. doi:10.31234/osf.io/xmpk4

Globig, L. K., Blain, B., & Sharot, T. (2020). *When Private Optimism meets Public Despair: Dissociable effects on behavior and well-being*. doi:10.31234/osf.io/gbdn8

Goh, J. X., Moran, J., Kerry, N., & Murray, D. (2020). *Outbreaks and outgroups: Three tests of the relationship between disease avoidance motives and xenophobia during an emerging pandemic*. doi:10.31234/osf.io/52bw4

González-Marrón, A., & Martínez-Sánchez, J. M. (2020). *Correlation between prevalence of tobacco smoking and risk and severity of COVID-19 at the national level in the European Union: an ecological study*. doi:10.1101/2020.04.28.20083352

Hania, A., & Hongbo, L. (2020). *PSYCHOLOGICAL PREDICTORS OF ANXIETY IN RESPONSE TO THE COVID-19 PANDEMIC: EVIDENCE FROM PAKISTAN*. doi:10.31234/osf.io/gk6mt

Harper, C. A., Satchell, L., Fido, D., & Latzman, R. D. (2020). *Functional fear predicts public health compliance in the COVID-19 pandemic*. doi:10.31234/osf.io/jkfu3

Hossain, M. A. (2020). *Is the spread of COVID-19 across countries influenced by environmental, economic and social factors?* doi:10.1101/2020.04.08.20058164

Hossain, A., Ali, M., Khan, H. R., & Ahsan, G. (2020). *Mental wellbeing in the Bangladeshi healthy population during nationwide lockdown over COVID-19: an online cross-sectional survey*. doi:10.1101/2020.05.14.20102210

Hyland, P., Shevlin, M., McBride, O., Murphy, J., Karatzias, T., Bentall, R., … Vallières, F. (2020). *Anxiety and depression in the Republic of Ireland during the COVID-19 pandemic*. doi:10.31234/osf.io/8yqxr

Imhoff, R., & Lamberty, P. (2020). *A bioweapon or a hoax? The link between distinct conspiracy beliefs about the Coronavirus disease (COVID-19) outbreak and pandemic behavior*. doi:10.31234/osf.io/ye3ma

Jarynowski, A., Wójta-Kempa, M., & Belik, V. (2020). *Trends in Perception of COVID-19 in Polish Internet*. doi:10.1101/2020.05.04.20090993

Kachanoff, F., Bigman, Y., Kapsaskis, K., & Gray, K. (2020). *Measuring Realistic and Symbolic Threats of COVID-19 and their Unique Impacts on Wellbeing and Adherence to Public Health Behaviors*. doi:10.31234/osf.io/5zr3w

Karwowski, M., Kowal, M., Groyecka, A., Bialek, M., Lebuda, I., Sorokowska, A., & Sorokowski, P. (2020). *When in Danger, Turn Right: Does Covid-19 Threat Promote Social Conservatism and Right-Wing Presidential Candidates?*doi:10.31234/osf.io/pjfhs

Kavanagh, N. M., Goel, R. R., & Venkataramani, A. S. (2020). *Association of County-Level Socioeconomic and Political Characteristics with Engagement in Social Distancing for COVID-19*. doi:10.1101/2020.04.06.20055632

Kazi, D. S., Wadhera, R. K., Shen, C., Ho, K. K. L., Patell, R., Selim, M. H., … Yeh, R. W. (2020). *Decline in Emergent and Urgent Care during the COVID-19 Pandemic*. doi:10.1101/2020.05.14.20096602

Kenyon, C. (2020). *Widespread use of face masks in public may slow the spread of SARS CoV-2: an ecological study*. doi:10.1101/2020.03.31.20048652

Kroencke, L., Geukes, K., Utesch, T., Kuper, N., & Back, M. (2020). *Neuroticism and Emotional Risk During the COVID-19 Pandemic*. doi:10.31234/osf.io/8c6nh

Kuper-Smith, B. J., Doppelhofer, L. M., Oganian, Y., Rosenblau, G., & Korn, C. (2020). *Risk perception and optimism bias during the early stages of the COVID-19 pandemic*. doi:10.31234/osf.io/epcyb

Kuratani, N. (2020). *Association of national Bacille Calmette-Guérin vaccination policy with COVID-19 epidemiology: an ecological study in 78 countries*. doi:10.1101/2020.05.13.20100156

Lan, F.-Y., Wei, C.-F., Hsu, Y.-T., Christiani, D. C., & Kales, S. N. (2020). *Work-related Covid-19 transmission*. doi:10.1101/2020.04.08.20058297

Leary, A., Dvorak, R., Leon, A. D., Peterson, R., & Troop-Gordon, W. (2020). *COVID-19 Social Distancing*. doi:10.31234/osf.io/mszw2

Leder, J., Pastukhov, A., & Schütz, A. (2020). *Even prosocially oriented individuals save themselves first: Social Value Orientation, subjective effectiveness and the usage of protective measures during the COVID-19 pandemic in Germany*. doi:10.31234/osf.io/nugcr

Li, J. B., Yang, A., Dou, K., & Cheung, R. Y. M. (2020). *Self-control moderates the association between perceived severity of the coronavirus disease 2019 (COVID-19) and mental health problems among the Chinese public*. doi:10.31234/osf.io/2xadq

Li, J. B., Yang, A., Dou, K., Wang, L.-X., Zhang, M.-C., & Lin, X. (2020). *Chinese public's knowledge, perceived severity, and perceived controllability of the COVID-19 and their associations with emotional and behavioural reactions, social participation, and precautionary behaviour: A national survey*. doi:10.31234/osf.io/5tmsh

Liang, J., Yuan, H.-Y., Wu, L., & Pfeiffer, D. U. (2020). *Estimating effects of intervention measures on COVID-19 outbreak in Wuhan taking account of improving diagnostic capabilities using a modelling approach*. doi:10.1101/2020.03.31.20049387

Malik, S. (2020). *Knowledge of COVID-19 Symptoms and Prevention among Pakistani Adults: A Cross-sectional Descriptive Study*. doi:10.31234/osf.io/wakmz

Malik, A. A., Couzens, C., & Omer, S. B. (2020). *COVID-19 related social distancing measures and reduction in city mobility*. doi:10.1101/2020.03.30.20048090

Marinthe, G., Brown, G., Delouvée, S., & Jolley, D. (2020). *Looking out for Myself: Exploring the Relationship Between Conspiracy Mentality, Perceived Personal Risk and COVID-19 Prevention Measures*. doi:10.31234/osf.io/cm9st

Mertens, G., Gerritsen, L., Duijndam, S., Salemink, E., & Engelhard, I. (2020). *Fear of the coronavirus (COVID-19): Predictors in an online study conducted in March 2020*. doi:10.31234/osf.io/2p57j

Messner, W., & Payson, S. E. (2020). *The Influence of Contextual Factors on the Initial Phases of the COVID-19 Outbreak across U.S. Counties*. doi:10.1101/2020.05.13.20101030

Moore, R. C., Lee, A., Hancock, J. T., Halley, M., & Linos, E. (2020). *Experience with Social Distancing Early in the COVID-19 Pandemic in the United States: Implications for Public Health Messaging*. doi:10.1101/2020.04.08.20057067

Mukherji, N. (2020). *The Social and Economic Factors Underlying the Incidence of COVID-19 Cases and Deaths in US Counties*. doi:10.1101/2020.05.04.20091041

Muto, K., Yamamoto, I., Nagasu, M., Tanaka, M., & Wada, K. (2020). *Japanese citizens' behavioral changes and preparedness against COVID-19: How effective is Japan's approach of self-restraint ?*doi:10.1101/2020.03.31.20048876

Naser, A. Y., Dahmash, E. Z., Al-Rousan, R., Alwafi, H., Alrawashdeh, H. M., Ghoul, I., … Dagash, A. (2020). *\textquotedblleftMental health status of the general population, healthcare professionals, and university students during 2019 coronavirus disease outbreak in Jordan: a cross-sectional study\textquotedblright*. doi:10.1101/2020.04.09.20056374

Okruszek, Ł., Aniszewska-Stańczuk, A., Piejka, A., Wiśniewska, M., & Żurek, K. (2020). *Safe but lonely? Loneliness, mental health symptoms and COVID-19*. doi:10.31234/osf.io/9njps

Oosterhoff, B., & Palmer, C. (2020). *Psychological Correlates of News Monitoring, Social Distancing, Disinfecting, and Hoarding Behaviors among US Adolescents during the COVID-19 Pandemic*. doi:10.31234/osf.io/rpcy4

Pana, T. A., Bhattacharya, S., Gamble, D. T., Pasdar, Z., Szlachetka, W. A., Perdomo-Lampignano, J. A., … Myint, P. K. (2020). *Country-level Determinants of the Severity of the First Global Wave of the COVID-19 Pandemic: An Ecological Study*. doi:10.1101/2020.05.13.20100677

Pennycook, G., McPhetres, J., Bago, B., & Rand, D. G. (2020). *Beliefs about COVID-19 in Canada, the U.K., and the U.S.A.: A novel test of political polarization and motivated reasoning*. doi:10.31234/osf.io/zhjkp

Pennycook, G., McPhetres, J., Zhang, Y., Lu, J. G., & Rand, D. G. (2020). *Fighting COVID-19 misinformation on social media: Experimental evidence for a scalable accuracy nudge intervention*. doi:10.31234/osf.io/uhbk9

Perrotta, D., Grow, A., Rampazzo, F., Cimentada, J., Fava, E. D., Gil-Clavel, S., & Zagheni, E. (2020). *Behaviours and attitudes in response to the COVID-19 pandemic: Insights from a cross-national Facebook survey*. doi:10.1101/2020.05.09.20096388

Pfattheicher, S., Nockur, L., Böhm, R., Sassenrath, C., & Petersen, M. B. (2020). *The emotional path to action: Empathy promotes physical distancing and wearing of face masks during the COVID-19 pandemic*. doi:10.31234/osf.io/y2cg5

Plohl, N., & Musil, B. (2020). *Modeling compliance with COVID-19 prevention guidelines: The critical role of trust in science*. doi:10.31234/osf.io/6a2cx

Raude, J., Debin, M., Souty, C., Guerrisi, C., Turbelin, C., Falchi, A., … Colizza, V. (2020). *Are people excessively pessimistic about the risk of coronavirus infection?* doi:10.31234/osf.io/364qj

Rentsch, C. T., Kidwai-Khan, F., Tate, J. P., Park, L. S., King, J. T., Skanderson, M., … Justice, A. C. (2020). *Covid-19 by Race and Ethnicity: A National Cohort Study of 6 Million United States Veterans*. doi:10.1101/2020.05.12.20099135

Rosenfeld, D. L. (2020). *Political Ideology and the Outbreak of COVID-19 in the United States*. doi:10.31234/osf.io/jrpfd

Rothgerber, H., Wilson, T., Whaley, D., Rosenfeld, D. L., Humphrey, M., Moore, A. L., & Bihl, A. (2020). *Politicizing the COVID-19 Pandemic: Ideological Differences in Adherence to Social Distancing*. doi:10.31234/osf.io/k23cv

Sala, G., Chakraborti, R., Ota, A., & Miyakawa, T. (2020). *Association of BCG vaccination policy and tuberculosis burden with incidence and mortality of COVID-19*. doi:10.1101/2020.03.30.20048165

Seale, H., Heywood, A. E., Leask, J., Sheel, M., Thomas, S., Durrheim, D. N., … Kaur, R. (2020). *COVID-19 is rapidly changing: Examining public perceptions and behaviors in response to this evolving pandemic*. doi:10.1101/2020.05.04.20091298

Siedner, M. J., Harling, G., Reynolds, Z., Gilbert, R. F., Haneuse, S., Venkataramani, A. S., & Tsai, A. C. (2020). *Social distancing to slow the U.S. COVID-19 epidemic: longitudinal pretest-posttest comparison group study*. doi:10.1101/2020.04.03.20052373

Simione, L., & Gnagnarella, C. (2020). *Differences between health workers and general population in risk perception, behaviors, and psychological distress related to COVID-19 spread in Italy*. doi:10.31234/osf.io/84d2c

Sorokowski, P., Groyecka-Bernard, A., Kowal, M., Sorokowska, A., Bialek, M., Lebuda, I., … Karwowski, M. (2020). *Can Information about Pandemics Increase Negative Attitudes toward Foreign Groups? A Case of COVID-19 Outbreak*. doi:10.31234/osf.io/j23vt

Šrol, J., Mikušková, E. B., & Cavojova, V. (2020). *When we are worried, what are we thinking? Anxiety, lack of control, and conspiracy beliefs amidst the COVID-19 pandemic*. doi:10.31234/osf.io/f9e6p

Stanley, M., Barr, N., Peters, K., & Seli, P. (2020). *Analytic-Thinking Predicts Hoax Beliefs and Helping Behaviors in Response to the COVID-19 Pandemic*. doi:10.31234/osf.io/m3vth

Sternisko, A., Cichocka, A., Cislak, A., & Van Bavel, J. J. (2020). *National Narcissism and the Belief and the Dissemination of Conspiracy Theories During the COVID-19 Pandemic: Evidence From 56 Countries*. doi:10.31234/osf.io/4c6av

Stults-Kolehmainen, M., Filgueiras, A., & Blacutt, M. (2020). *Factors linked to changes in mental health outcomes among Brazilians in quarantine due to COVID-19*. doi:10.1101/2020.05.12.20099374

Sweeny, K., Rankin, K., Cheng, X., Hou, L., Long, F., Meng, Y., … Zhang, W. (2020). *Flow in the Time of COVID-19: Findings from China*. doi:10.31234/osf.io/e3kcw

Tabri, N., Hollingshead, S., & Wohl, M. J. A. (2020). *Framing COVID-19 as an Existential Threat Predicts Anxious Arousal and Prejudice towards Chinese People*. doi:10.31234/osf.io/mpbtr

Teovanovic, P., Lukic, P., Zupan, Z., Lazić, A., Ninković, M., & Zezelj, I. (2020). *Irrational beliefs differentially predict adherence to guidelines and pseudoscientific practices during the COVID-19 pandemic*. doi:10.31234/osf.io/gefhn

Vachuska, K. F. (2020). *Initial Effects of the Coronavirus Pandemic on Racial Prejudice in the United States: Evidence from Google Trends*. doi:10.31235/osf.io/bgpk3

Vega, R. D. L., Barquin, R. R., Boros, S., & Szabo, A. (2020). *Could Attitudes Toward COVID-19 in Spain Render Men More Vulnerable Than Women?* doi:10.31234/osf.io/dyxqn

Westgard, B. C., Morgan, M. W., Vazquez-Benitez, G., Erickson, L. O., & Zwank, M. D. (2020). *Characteristics of patients presenting, and not presenting, to the emergency department during the early days of COVID-19*. doi:10.1101/2020.05.05.20090795

Wise, T., Zbozinek, T. D., Michelini, G., Hagan, C. C., & Mobbs, D. (2020). *Changes in risk perception and protective behavior during the first week of the COVID-19 pandemic in the United States*. doi:10.31234/osf.io/dz428

Wissmath, B., Mast, F. W., Kraus, F., & Weibel, D. (2020). *Understanding the psychological impact of the COVID-19 pandemic and containment measures: an empirical model of stress*. doi:10.1101/2020.05.13.20100313

Yang, J., Gong, H., Chen, X., Chen, Z., Deng, X., Qian, M., … Yu, H. (2020). *Health seeking behaviors of patients with acute respiratory infections during the outbreak of novel coronavirus disease 2019 in Wuhan, China*. doi:10.1101/2020.05.05.20091553

Zettler, I., Schild, C., Lilleholt, L., Kroencke, L., Utesch, T., Moshagen, M., … Geukes, K. (2020). *The role of personality in COVID-19 related perceptions, evaluations, and behaviors: Findings across five samples, nine traits, and 17 criteria*. doi:10.31234/osf.io/pkm2a

Zheng, M. X., Yao, J., & Narayanan, J. (2020). *Mindfulness Buffers the Impact of COVID-19 Outbreak Information on Sleep Duration*. doi:10.31234/osf.io/wuh94

Supplementary Materials 6

Questions used in the repliCATS process for eliciting judgements about replicability

Table SM6. Questions used in the repliCATS process for eliciting judgements about replicability

**Round 1 elicitation questions**			
Question		Response format	Platform guidance (tooltip)
1	How well do you understand this claim?	7-point scale with a free-text box for comments [You can restate the claim in clearer terms here. You could also highlight terms or concepts that you didn't understand. You have limited space to do this here, but don't worry, in question 4 below you can tell us any other thoughts you may have.]	We know the clarity and comprehensibility of scientific papers varies. We are interested in your honest account of how well you understand this claim.
2	What's your initial reaction: is the underlying effect or relationship plausible?	Binary Yes/No	We know this is a broad question. We're trying to get a sense of your intuition about whether the effect or relationship underlying the claim is real, regardless of its exact operationalisation in this study. For example, would you give it a high prior probability?
3	What is the probability that direct replications of this study would find a statistically significant effect in the same direction as the original claim (0-100%)? 0 means that you think that a direct replication would never succeed, even by chance. 100 means that you think	Three-point elicitation with a free-text box for comments [What factors influenced your judgement? What, if anything, made you think that it might successfully replicate? What, if anything, made you think that it might not successfully replicate?]	To answer this question imagine 100 replications of the original study, combined to produce a single, overall replication estimate (e.g., a meta-analysis with no publication bias). How likely is it that the overall estimate will be similar to the original? Note that all

	that a direct replication would never fail, even by chance.		replication studies are 'direct' replications, i.e., they constitute reasonable tests of the original claim, despite minor changes that may have occurred in methods or procedure. And all replication studies have high power (90% power to detect an effect 50-75% of the original effect size with alpha=0.05, two-sided). For your lower bound, think of factors that make successful replications unlikely. For your upper bound, think of factors that make successful replications likely. For your best estimate, consider the balance of factors.
4	Considering the major factors that influenced your thinking in making these judgements, please describe any important aspects that you have not covered above.	Free-text box for comments [You should answer this freely. You might consider describing your initial impressions of the article, any personal experience you have with such claims, why you found the claim plausible/implausible, how familiar you are with similar studies, or unusual terms or concepts.]	We are interested in identifying what aspects most influenced your decision making processes. Please share any aspects that were important, but which were not included in the comments above.
5	Were you involved in the writing, data collection, or analysis of the original study?	Binary Yes/No	Were you an author of, or involved in any aspect of the writing, data collection or analysis, for this original study?
**Round 2 elicitation questions**			
1	Now that you've discussed this claim with your group, how well do you	7-point scale with a free-text box for comments [If the discussion has changed your understanding	Same as Round 1

	understand it?	of what this claim means, please briefly state what your new understanding is.]	
2	Now that you've discussed this claim with your group, do you consider the underlying effect or relationship to be plausible?	Binary Yes/No	Same as Round 1
3	What is the probability that direct replications of this study would find a statistically significant effect in the same direction as the original claim (0-100%)? 0 means that you think that a direct replication would never succeed, even by chance. 100 means that you think that a direct replication would never fail, even by chance.	Three-point elicitation with a free-text box for comments [What factors influenced your judgement? What, if anything, made you think that it might successfully replicate? What, if anything, made you think that it might not successfully replicate?]	Same as Round 1
4	What factors were important in rethinking your judgements?	Free-text box for comments [You should answer this freely. You might describe the most relevant, important or interesting aspects of the discussion, and how these influenced your Round 2 judgements. This comment will not be displayed. If you have information or thoughts about this claim that you were not comfortable sharing with the group, this is an opportunity to include them.]	We are interested in which aspects of the discussion most influenced your perspective or level of uncertainty, particularly those that may have changed your initial judgements.
