# Supplementary material for: Predicting the replicability of social and behavioural science claims in COVID-19 preprints
Source: Nat Hum Behav. 2024 Dec 20;9(2):287–304. doi: 10.1038/s41562-024-01961-1 (PMC11860236; doi:10.1038/s41562-024-01961-1)
Supplement: Supplementary file 2 — Reporting Summary [file 41562_2024_1961_MOESM2_ESM.pdf]

nature portfolio

Corresponding author(s): Alexandru Marcoci

Last updated by author(s): Jun 18, 2024

Reporting Summary

Nature Portfolio wishes to improve the reproducibility of the work that we publish. This form provides structure for consistency and transparency in reporting. For further information on Nature Portfolio policies, see our Editorial Policies and the Editorial Policy Checklist.

Statistics

For all statistical analyses, confirm that the following items are present in the figure legend, table legend, main text, or Methods section.

n/a	Confirmed	
☐	☒	The exact sample size (*n*) for each experimental group/condition, given as a discrete number and unit of measurement
☐	☒	A statement on whether measurements were taken from distinct samples or whether the same sample was measured repeatedly
☐	☒	The statistical test(s) used AND whether they are one- or two-sided<br>*Only common tests should be described solely by name; describe more complex techniques in the Methods section.*
☐	☒	A description of all covariates tested
☐	☒	A description of any assumptions or corrections, such as tests of normality and adjustment for multiple comparisons
☐	☒	A full description of the statistical parameters including central tendency (e.g. means) or other basic estimates (e.g. regression coefficient) AND variation (e.g. standard deviation) or associated estimates of uncertainty (e.g. confidence intervals)
☐	☒	For null hypothesis testing, the test statistic (e.g. *F*, *t*, *r*) with confidence intervals, effect sizes, degrees of freedom and *P* value noted<br>*Give P values as exact values whenever suitable.*
☒	☐	For Bayesian analysis, information on the choice of priors and Markov chain Monte Carlo settings
☐	☒	For hierarchical and complex designs, identification of the appropriate level for tests and full reporting of outcomes
☐	☒	Estimates of effect sizes (e.g. Cohen's *d*, Pearson's *r*), indicating how they were calculated

Our web collection on statistics for biologists contains articles on many of the points above.

Software and code

Policy information about availability of computer code

Data collection	All scripts are available for the structured groups (https://osf.io/4sfbj/) and for the replication markets (http://osf.io/5kfc6/).
Data analysis	All analyses were conducted using R v4.3.1 and the code is available at osf.io/4sfbj/

For manuscripts utilizing custom algorithms or software that are central to the research but not yet described in published literature, software must be made available to editors and reviewers. We strongly encourage code deposition in a community repository (e.g. GitHub). See the Nature Portfolio guidelines for submitting code & software for further information.

Data

Policy information about availability of data

All manuscripts must include a data availability statement. This statement should provide the following information, where applicable:

- Accession codes, unique identifiers, or web links for publicly available datasets
- A description of any restrictions on data availability
- For clinical datasets or third party data, please ensure that the statement adheres to our policy

The full datasets of claim content, metadata for the 100 COVID-19 preprints, and the full dataset of all replication outcomes, including preregistrations and OSF projects, are available at https://doi.org/10.17605/OSF.IO/FJKSB.
The judgement elicitation datasets are available for the structured groups (https://osf.io/4sfbj/) and for the replication markets (http://osf.io/5kfc6/).

Research involving human participants, their data, or biological material

Policy information about studies with human participants or human data. See also policy information about sex, gender (identity/presentation), and sexual orientation and race, ethnicity and racism.

Reporting on sex and gender	Information on repliCATS participant gender was collected using a survey, but this field was optional. Gender was not analysed directly and only used to show demographic characteristics of the expert and novice groups. Individual-level data on gender is not made publicly available.
Reporting on race, ethnicity, or other socially relevant groupings	We do not report on race, ethnicity or any other similar groupings.
Population characteristics	repliCATS collected demographic variables of participants (including age, education level) but they were not directly analysed and only used to show demographics characteristics of the expert and novice groups.
Recruitment	In order to test the predictive ability of participants with varying levels of task expertise, we recruited:<br><br>- 99 experts from a pool of people who had previously participated in at least one repliCATS workshop or remote process for evaluating research claims, described in Fraser et al. (2023). Each expert participant was awarded a $200USD grant to assess 10 research claims for this study.<br><br>- 96 novices from three undergraduate courses (ranging from an introductory course to a capstone one) taught by AM at the University of North Carolina, Chapel Hill. Eighty-eight novices completed their assessments and only their data has been included in the analyses. Novice participants were offered 3 extra credits in their respective courses to assess the replicability of 10-13 claims.<br><br>The sample is not representative.
Ethics oversight	Replications. The procedures were approved by the local ethics review board at each institution that conducted replications with concurrence from the United States Army Medical Research and Development Command's Office of Research Protections, Human Research Protection Office (HRPO) or the United States Naval Information Warfare Center Pacific, HRPO.<br><br>Judgement elicitation (structured groups). The procedures were approved by the University of Melbourne [#1853445.6] and the University of North Carolina, Chapel Hill's Office of Human Research Ethics [#19-3104].<br><br>Judgement elicitation (replication markets). The procedures were approved by the Harvard University CUHS [#18-1729].

Note that full information on the approval of the study protocol must also be provided in the manuscript.

Field-specific reporting

Please select the one below that is the best fit for your research. If you are not sure, read the appropriate sections before making your selection.

☐ Life sciences ☒ Behavioural & social sciences ☐ Ecological, evolutionary & environmental sciences

For a reference copy of the document with all sections, see nature.com/documents/nr-reporting-summary-flat.pdf

Behavioural & social sciences study design

All studies must disclose on these points even when the disclosure is negative.

Study description	All analyses are quantitative and fit with either linear mixed effects models or simple correlations. There are some basic qualitative descriptors of the demographic characteristics of the participant pool.
Research sample	We recruited 99 experts from a pool of people who had previously participated in at least one repliCATS workshop or remote process for evaluating research claims, described in Fraser et al. (2023). Each expert participant was awarded a $200USD grant to assess 10 research claims for this study.<br><br>The 96 novices were recruited from three undergraduate courses (ranging from an introductory course to a capstone one) taught by AM at the University of North Carolina, Chapel Hill. Eighty- eight novices completed their assessments and only their data has been included in the analysis. Novice participants were offered 3 extra credits in their respective courses to assess the replicability of 10-13 claims.
Sampling strategy	The study sample was chosen such that we achieved the minimum number of assessments required per claim assessed from each group without providing too much of a cognitive burden on any given participant. The expert and novice group population samples are representative of people with and without experience assessing the replicability of scientific evidence.
Data collection	Expert elicitation data was collected using a custom-built online platform (described in Pearson et al. 2021). There was not anyone

Data collection	present besides the participants and members of our team who were facilitating the discussion. The researchers were not blind to the experimental condition or preregistered hypotheses during data collection.
Timing	Data collection took place from 28th August to 17th September 2020 for experts (experienced participants), and in two 3-week waves, in the Fall 2020 and Spring 2021 semesters for novices (beginner participants).
Data exclusions	We excluded data collected from one participant in the expert group as their highest level of completed education was high school. This was a pre-registered exclusion criteria. We also excluded 6 research claims from the results reported in the Main Text following reviewer 3's comments about low power. This was not a pre-registered exclusion criterion, so we include all results reported in the main text on the full dataset of 35 research claims as supplementary materials. Following a second round of reviews and subsequent conversations about how power should be computed we decided to include in the Discussion section and as an additional appendix a report of all our results from the Main Text for a sub-corpus 24 research claims (which excludes a further 5 claims). These exclusions are transparent and justified in the text.
Non-participation	Eight participants from the novice group signed up to participate but did not complete their assigned assessments and did not contribute any data used in the analysis.
Randomization	Participants were assigned to expert (experienced participants) or novice (beginner participants) groups based on their prior experience assessing the replicability of scientific evidence. Within these experimental groups participants were randomly allocated into smaller groups of 4-6 participants to assess any given piece of evidence.

Reporting for specific materials, systems and methods

We require information from authors about some types of materials, experimental systems and methods used in many studies. Here, indicate whether each material, system or method listed is relevant to your study. If you are not sure if a list item applies to your research, read the appropriate section before selecting a response.

Materials & experimental systems

n/a	Involved in the study
☒	☐ Antibodies
☒	☐ Eukaryotic cell lines
☒	☐ Palaeontology and archaeology
☒	☐ Animals and other organisms
☒	☐ Clinical data
☒	☐ Dual use research of concern
☒	☐ Plants

Methods

n/a	Involved in the study
☒	☐ ChIP-seq
☒	☐ Flow cytometry
☒	☐ MRI-based neuroimaging

Plants

Seed stocks	*Report on the source of all seed stocks or other plant material used. If applicable, state the seed stock centre and catalogue number. If plant specimens were collected from the field, describe the collection location, date and sampling procedures.*
Novel plant genotypes	*Describe the methods by which all novel plant genotypes were produced. This includes those generated by transgenic approaches, gene editing, chemical/radiation-based mutagenesis and hybridization. For transgenic lines, describe the transformation method, the number of independent lines analyzed and the generation upon which experiments were performed. For gene-edited lines, describe the editor used, the endogenous sequence targeted for editing, the targeting guide RNA sequence (if applicable) and how the editor was applied.*
Authentication	*Describe any authentication procedures for each seed stock used or novel genotype generated. Describe any experiments used to assess the effect of a mutation and, where applicable, how potential secondary effects (e.g. second site T-DNA insertions, mosiacism, off-target gene editing) were examined.*
